# Mediastinoscope-controlled parasternal fenestration of the pericardium: definitive surgical palliation of malignant pericardial effusion

**DOI:** 10.1186/1749-8090-7-56

**Published:** 2012-06-19

**Authors:** Imre Toth, Geza Szucs, Tamas F Molnar

**Affiliations:** 1 Semmelweis Teaching Hospital, Department of General and Thoracic Surgery, Csabai kapu 9.-11, H-3529, Miskolc, Hungary; 2 University of Pecs, Medical School, Surgery Clinic, Szigeti ut 12, H-7624, Pecs, Hungary

**Keywords:** Pericardial fenestration, Pericardial effusion, Malignant effusion

## Abstract

**Background:**

The tumorous infiltration or carcinosis of the pericardium could cause pericardial effusion in up to one-third of cases of malignancy, thus potentially interfere with the otherwise desirable oncological treatment. The existing surgical methods for the management of pericardial fluid are well-established but are not without limitations in the symptomatic relief of malignant pericardial effusion (MPE). The recurrence rate ranges between 43 and 69% after pericardiocentesis and 9 to 16% after pericardial drainage. The desire to overcome relative limitations of the existing methods led us to explore an alternative approach.

**Methods:**

The standard armamentarium of the Carlens collar mediastinoscopy procedure was utilized in a Chamberlain parasternal approach of the pericardial sac. The laterality of approach was decided based upon the pleural involvement, as tumor-free pericardiopleural reflection is required. A pericardio-pleural window at least 3 cm in diameter was created. From January 2000 to December 2009, 22 cases were operated on with mediastinoscope-controlled parasternal fenestration (MCPF). Considering the type of the primary tumor, there were 11 lung cancer, 6 breast cancers, 2 haematologic malignancies and in 3 patients the origin of malignancy could not be verified.

**Results:**

There were no operative deaths. We lost one patient (4.5%) in the postoperative hospital period. All of the surviving patients had a minimum of 2 months of symptom-free survival. We detected transient recurrence of MPE in one patient (4.5%) 14 days after the MCPF, which disappeared spontaneously after 24 hours.

**Conclusion:**

The MCPF offers a real alternative in certain cases of pericardial effusion. We recommend this method especially for the definitive surgical palliation of MPE.

## Background

The number of patients with secondary malignant pericardial effusion is steadily increasing due to the improving efficacy of complex cancer treatment modalities. Metastases to the myocardium have reportedly been found in up to 1.23% of all autopsies [1]. The incidence of malignancy associated with pericardial involvement ranges from 8 to 20% in other autopsy studies [2-4]. The tumorous infiltration or carcinosis of the pericardium could cause pericardial effusion in up to one-third of cases of malignancy, thus potentially interfere with the otherwise desirable oncological treatment [4].

The existing surgical methods for the management of pericardial fluid are well-established but are not without limitations in providing symptomatic relief of malignant pericardial effusion. Pericardiocentesis and the Larrey-Fontenelle approaches [5,6] using a subxyphoideal fenestration have a recurrence rate ranging between 43 and 69% after pericardiocentesis [3,7] and 9 and 16% after pericardial drainage [8-11]. The video-assisted thoracoscopic surgical (VATS) pericardio-pleural fenestration is highly efficient but requires anaesthesia with contralateral single-lung ventilation which is compromising in patients with an already reduced cardio-pulmonary reserve [8,12,13]. The transdiaphragmatic pericardial fenestration was developed as an alternative method, but the pericardio-peritoneal window might be blocked by the adjacent abdominal structures and is a two-cavity procedure [14,15]. The desire to overcome the relative limitations detailed above led us to explore an alternative approach.

Having extensive experience in collar mediastinoscopy, we utilized the existing armamentarium of the Carlens procedure [16] in a Chamberlain approach in order to achieve pericardial decompression in the form of a pericardiopleural window [17-19].

## Methods

**Figure 1:**
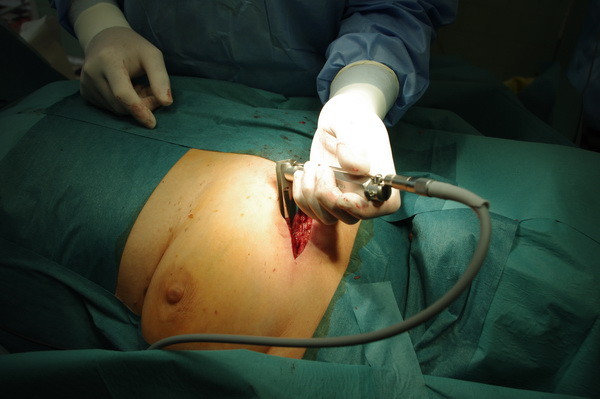
The position of patient and the place of incision of left side mediastinoscope-controlled parasternal pericardial fenestration.

**Figure 2:**
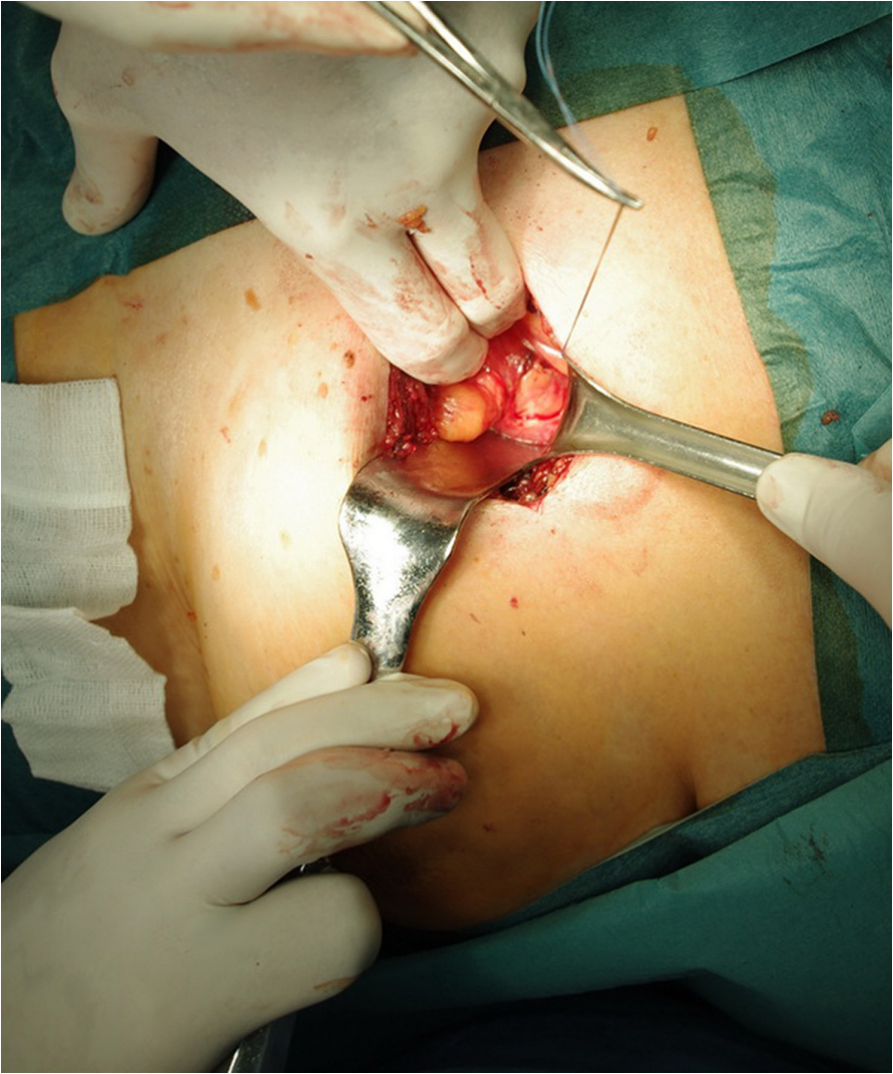
The place of pericardial window (left side).

**Figure 3:**
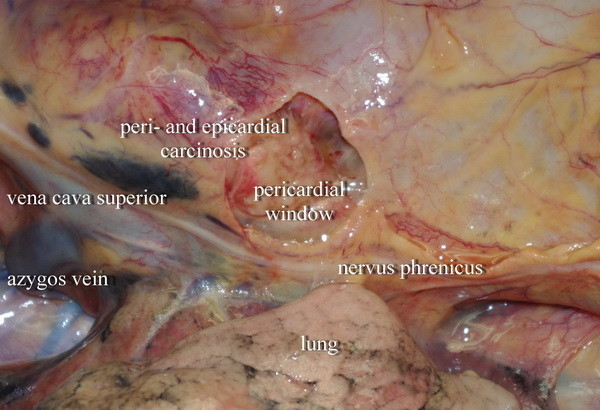
The intrapleural situation after parasternal pericardial fenestration (right side).

The patient was placed in a 10–30 degree supine Fowler position. Anaesthesia was introduced with single lumen endotracheal tube. A transverse skin incision was made above the sternal end of the third or fourth rib, a little bit longer than the expected length of its cartilaginous part. The laterality of approach (i.e. left or right) was decided based upon the pleural involvement, as tumor-free pericardiopleural reflection is required. In the case of synchronous pleural carcinosis the opposite side was our choice. A left-sided approach was preferred when both pleural cavities were suitable for the procedure. After blind splitting the pectoral muscles the sternal edge of the rib was resected. The internal thoracic artery and vein were identified and secured in order to avoid annoying bleeding in the course of later manipulation. The pleural cavity was opened close to its mediastinal reflection. A limited exploration of the thoracic cavity was undertaken introducing a rigid mediastinoscope (Karl Storz GMBH, Tuttlingen, Germany), and biopsies were performed if suspicious parenchyma/pleural lesions were present (Figure 1). Keeping aside the lung with a long retractor a holding stitch secured pericardial access ventrally to the phrenic nerve. Lifting up this stitch, a window an at least 3 cm in diameter was created on the pericardium using scissors to manipulate within or parallel to the main tube of the mediastinscope (Figure 2, 3). The effluent pericardial liquid was emptied with mild suction and a 20 Ch marked silicone chest tube was inserted into the pleural cavity. The chest tube was kept under suction (50–100 mbar) for 48 to 72 hours and was removed following chest X-ray control. Bacterial and cytological examination of the pericardial fluid and the histological examination of the pericardium specimen completed the procedure. The pain in the first postoperative days was alleviated with parenteral Tramadol and Diclofenac administration.

From January 2000 to December 2009, 22 cases out of the 73 consecutive patients with pericardial fluid that we treated were operated on with the mediastinoscope-controlled parasternal pericardial fenestration (MCPF) described above. 12 female and 10 male with average age of 57 years (min. 26, max. 72, SD 11) underwent MCPF. Almost all the MCPF cases were operated on by two of the six surgeons in the department. So, in the first part of the examined period the patient selection for MCPF depended primarily on the surgeon. In the second half of the examined period, we preferred to perform MCPF in cases of MPE if the patient had no severe symptoms of pericardium tamponade. If so we were compelled to perform percutaneous pericardiocentesis or subxyphoideal fenestration. In non-malignant cases also the subxyphoideal fenestration is preferred. The methods other than MCPF that we applied and the number of patients are summarized in Table 1.

**Table 1:** Different surgical methods for solving pericardial effusion in the examined period

	
Percutaneous pericardiocentesis + drain	23
Subxyphoideal fenestration	23
**Mediastinoscopy-controlled parasternal fenestration**	**22**
Transdiaphragnatic pericardio-peritoneal fenestration	2
VATS pericardio-pleural fenestration	2
Thoracotomy, pericardio-pleural fenestration	1
**Total**	**73**

The average procedure time of MCPF was 31 minutes (min. 20, max. 50, SD 9,5) and the average hospital stay was 9.4 days (min. 7, max. 11, SD 1,3). Considering the type of the primary tumor, there were 11 lung cancer, 6 breast, no gastrointestinal, 2 haematologic malignancies. In 3 cases out of 22 parasternally fenestrated patients, a malignant origin was not verified.

**Figure 4:**
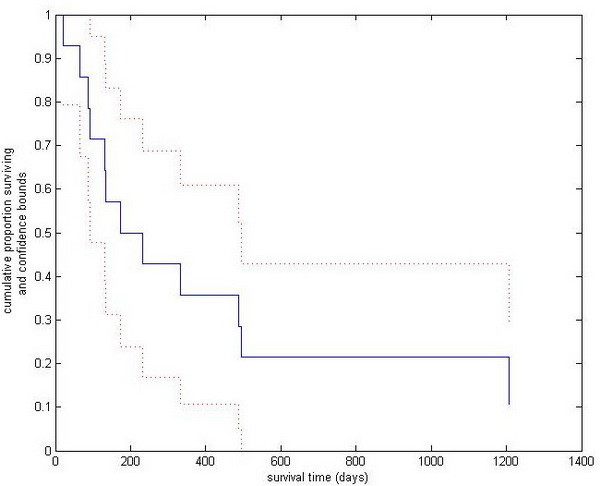
The cumulative distribution of patients survival, and the lower and upper confidence bounds of cumulative distribution of survival after parasternal pericardial fenestration.

For statistical analysis we used the “Statistica 9.0” software package (Stat Soft Inc. USA). The mean values and the standard deviation (SD) were calculated for age, operation time, hospital stay and survival. The confidence interval was 95%. The Kaplan-Meier survival curve was made also with the Statistica 9.0 software package. The result was controlled with the “Matlab R2010b” software package (MathWorks Inc. USA). This calculation also shows the lower and upper confidence bounds for the cumulative distribution of survival (Figure 4). These bounds were calculated using Greenwood’s formula. In the cases of those two patients who were still alive at the end of the examined period, the survival times were used in the calculation and are shown in the curve as censored data. The comparison of survivals was analyzed with Student’s two-sample *t*-test. The critical value of “t” was given at a level of 95% (significance level: p < 0.05) with n1 + n2-2 degrees of freedom and was compared with the calculated “t” value. The statistical analyses were calculated with an assumed normal distribution. Due to the low number of patients, the result is only of informative value and would require an even larger test sample.

## Results

There were no operative deaths in the group of 22 MCPF patients. Transient intraoperative dysrhythmia was detected in 3 cases (14%). We lost one patient (4.5%) in the postoperative hospital stay. This end-stage cachectic lung-cancer patient died due to a cascade of inflammations. The mean hospital stay of our patients was 9.4 days (SD 1.3).

The postoperative pain was easily relievable in spite of the small partial subperiosteal parasternal rib resection. Usually after two to four days our patients did not require additional continuous therapy with painkillers because of postoperative pain. Our patients recovered without difficulty – considering their original complaints and physical incapacity caused by their malignant disease.

Due to the low number and the heterogeneity of the reported 22 MCPF patients regarding their origin of primary malignancy and performance status, the long-term survival rate could be only of informative value (Figure 4). The follow-ups and the oncologic treatment were done in different hospitals and not by us. We had correct information regarding survival for our MCPF patients in 14 cases of 22. All of the surviving patients had a minimum of 2 months of symptom-free survival. The detailed data of our MCPF patients can be seen in Table 2.

**Table 2:** The demography, primary malignancy, operative time, hospital stay and the survival of the 22 patients operated with MCPF

**No**	**Age**	**Sex**	**Primary malignancy**	**Op time (min.)**	**Hosp stay (days)**	**Survival (days)**	**Status 2011.05.31.**
1	51	m	pulmonary	20	11	131	
2	53	m	pulmonary	20	10	487	
3	68	f	pulmonary	30	10	?	
4	53	f	breast	35	11	1207	
5	50	f	?	50	9	?	
6	46	f	breast	25	8	?	
7	47	m	pulmonary	40	7	20	
8	72	f	?	40	9	1275	alive
9	50	f	breast	40	10	135	
10	47	m	pulmonary	25	8	?	
11	65	m	pulmonary	35	10	93	
12	71	f	hematologic	50	10	495	
13	57	f	breast	40	9	334	
14	54	m	?	20	10	65	
15	26	m	pulmonary	20	11	231	
16	60	m	pulmonary	40	10	88	
17	73	f	breast	20	10	730	alive
18	59	m	pulmonary	30	11	173	
19	67	f	pulmonary	30	7	?	
20	61	f	breast	25	7	?	
21	68	f	hematologic	30	9	?	
22	55	m	pulmonary	25	10	?	
**Mean**	**57**			**31**	**9.4**	**390**	
SD	11			9.5	1.3	413	
			**mean pulmonary**	**28.6**	**9.5**	175*	
			SD pulmonary	7.4	1.5	153	
			**mean breast**	**30.8**	**9.2**	601*	
			SD breast	8.6	1.5	473	

*Statistically significant difference, p < 0.05.

We detected transient recurrence of the pericardial fluid in one patient (4.5%) 14 days after the MCPF. The echocardiography revision after 24 hours did not detect an echolucent zone around the heart, and the recidive pericardial effusion disappeared spontaneously.

## Discussion

The demand for a definitive palliative treatment of neoplastic pericardial effusion led us to develop an alternative method of pericardial fenestration.

The aim of the present retrospective study was to publish the methodology of mediastinoscope-controlled parasternal fenestration of the pericardium (MCPF) that we have developed and applied. The pericardio-pleural fenestration with parasternal approach is an easy to perform, straightforward procedure. It is easy demonstrable, trainee-friendly, and does not require sophisticated manual skill. It requires no special tools. Single-lung ventilation is not necessary in the course of the operation. This strictly palliative method is minimally invasive, cost efficient, and provides immediate relief from complaints and symptoms caused by the pericardial effusion. The pericardial fluid produced empties into the pleural cavity across the pericardio-pleural window and will be absorbed on the bigger pleural surface. In our 10 year series of operations with 22 patients there was no prolonged recurrence of pericardial effusion, so the MCPF fulfilled what we had expected with a low hospital mortality rate.

Unfortunately, the idea presented is not exclusively new. A method not significantly different from ours was first described by Calvin in 1971. He suggested the parasternal mediastinotomy as a possible clinical application for pericardial fenestration [20]. We have found one article of a case report using the same approach for solving the problem of tension pneumo-pericardium [21] and another about pericardioscopy performed with the same armamentarium [22]. However our Pub Med search was unable to reveal any publication that discussed an application of parasternal pericardial fenestration in cases of neoplastic pericardial effusion.

## Conclusions

The existing methods for solving the clinical consequences of pericardial fluid are well-known and safely performed. We do not offer our method instead of them!

However the malignant pericardial effusion and its relatively high recurrence rate is a special problem. The MCPF is a possible choice in such special cases. The mortality was low and the recurrence rate tended to zero in our patients treated with MCPF. Single-lung ventilation was not necessary in the course of the operation. The postoperative care and the mobilization of our patients were without difficulty and the postoperative pain was easily relievable in spite of the small partial subperiosteal parasternal rib resection. In our everyday practice we definitely prefer MCPF in cases of MPE to VATS or subxyphoideal fenestration.

On the other hand we do not think it a mistake to perform MCPF in non-malignant cases, but considering its advantages we recommend this “new-old method” especially for the definitive surgical palliation of pericardial effusion with malignant origin.

## Abbreviations

Ch, Charriere, 0,33 mm; MCPF, Mediastinoscope-controlled parasternal pericardial fenestration; MPE, Malignant pericardial effusion; SD, Standard deviation; VATS, Video-assisted thoracoscopic surgery.

## Authors’ contributions

IT: Made substantial contributions to conception and design, or acquisition of data, drafted and translated the manuscript from Hungarian into English. Performed or assisted almost 50% of operations of the study. Looked for the literature of the theme. Performed the statistical analysis. GSz: Participated in the design of the study, revised it critically for important intellectual content. Performed or assisted almost 50% of operations of the study. Performed the first MCPF in the department. TFM: Conceived of the study, criticised, corrected it. Helped to draft the manuscript with his advices and his experiences as he had published before in this theme (pericardio-peritoneal shunt). All authors read and approved the final manuscript.

## Authors’ information

IT: MD, chief consultant surgeon and consultant general thoracic surgeon in Semmelweis Teaching Hospital, Miskolc, Hungary, GSz: MD, PhD, head of the Department of General and Thoracic Surgery, Semmelweis Teaching Hospital, Miskolc, Hungary, TFM: MD, PhD, professor, head of the Department of Thoracic Surgery, University of Pecs, Surgery Clinic, Pecs, Hungary.
